# MAGIN-GO: Protein function prediction based on dual graph neural networks and gene ontology structure

**DOI:** 10.1371/journal.pone.0342072

**Published:** 2026-02-09

**Authors:** Runxin Li, Wentao Xie, Zhenhong Shang, Xiaowu Li, Guofeng Shu, Lianyin Jia, Wei Peng

**Affiliations:** 1 Yunnan Key Laboratory of Computer Technologies Application, Kunming University of Science and Technology, Kunming, China; 2 Faculty of Information Engineering and Automation, Kunming University of Science and Technology, Kunming, China; Old Dominion University, UNITED STATES OF AMERICA

## Abstract

Proteins are fundamental to the execution of biological activities, and the accurate prediction of their functions is of paramount importance for protein research. Recent advancements in deep learning, particularly those based on Graph Neural Networks (GNNs), have demonstrated promising results by integrating protein graph features with sequence information. However, traditional GNN methods exhibit limitations in their feature representation capabilities, failing to capture long-range dependencies within sequences and lacking incorporation of inter-annotation relationships. To address these challenges, we propose a method, MAGIN-GO, which combines Graph Isomorphism Network (GIN) and Graph Convolutional Network (GCN) with Graph Convolutional Self-Attention Network (GMSA) to extract multi-source protein information and integrates Gene Ontology (GO) annotation embeddings. Our method effectively combines protein sequence features with protein-protein interaction (PPI) graph node features, extracts topological and contextual information through GIN and GMSA, and integrates pre-trained GO term embeddings into a multi-label classification framework. Comprehensive experiments on the UniProtKB/Swiss-Prot dataset demonstrate that MAGIN-GO outperforms existing methods, achieving AUPR values of 0.569, 0.434, and 0.754 for Molecular Function (MF), Biological Process (BP), and Cellular Component (CC) domains, respectively, with corresponding Fmax scores of 0.568, 0.458, and 0.752, Smin scores of 11.297, 37.709, and 8.079, and AUC scores of 0.896, 0.897, and 0.940. The experimental results showed that the performance of MAGIN-GO was good and superior to the existing methods.

## Introduction

Proteins, as the expression products of genes and essential macromolecules in living organisms, constitute the fundamental material basis for life activities. They participate in a broad spectrum of essential biological processes, including signal transduction, the catalysis of metabolic reactions, and the maintenance of cellular structure. As indispensable components of living systems, proteins play crucial roles in diverse biological functions. The study of proteins holds significant practical importance for identifying drug targets, elucidating disease mechanisms, and advancing biotechnological applications [1].

In recent years, high-throughput sequencing technology has advanced continuously, leading to a rapid increase in protein data. However, only a small number of proteins have been functionally annotated. For example, in the Universal Protein UniProt (UniProt Consortium 2018) database [2], more than 100,000 proteins have been obtained through biological experiments with standard functional annotations. This represents only 0.1 of the proteins in the UniProt database. However, the traditional wet-lab methods for verifying protein function are time-consuming and labor-intensive, with stringent requirements for experimental equipment and funding, making them insufficient to meet the growing demand for protein function annotation [3]. Therefore, the development of efficient computational approaches for protein function prediction is highly desirable.

Since proteins possess multiple functions, the protein function prediction problem can be regarded as a multi-label classification problem, i.e., by extracting the features of a given protein and mapping them to the protein function label space. Protein functions are described by Gene Ontology (GO) [4], which is one of the most successful ontologies in biology. GO framework is organized into three distinct sub-ontologies based on the scope of function: Molecular Function Ontology (MF), Biological Process Ontology (BP), and Cellular Component Ontology (CC). Protein functions are experimentally determined by researchers and subsequently documented in reports. These annotations are then incorporated into the knowledge base by database administrators and are often propagated to homologous proteins.

In general, the study of protein function prediction can be divided into three stages. The first stage is the sequence-based methods, such as BLAST [5], which searches for homologous sequences of a target protein with a known function by sequence comparison and then transfers its known function to the target protein. The limitations of this approach are that it is difficult to predict proteins without sequence similarity and it is time consuming to determine the function of the protein. The second stage involves prediction based on traditional machine learning approaches. For instance, the multi-source k-nearest neighbor (k-NN) [6] algorithm is employed, which integrates multiple similarity calculation methods to identify the k nearest neighbors of the target protein. The current annotation of the predicted protein is then determined by calculating the weighted average of the functions of its nearest neighbor proteins. However, most machine learning-based prediction methods rely on manually extracted protein sequence features, which can result in substantial computational overhead for the prediction process. The third stage is prediction based on deep learning methods. In 2018, DeepGO [7] was the first method to apply deep learning to protein function prediction. The method learns features from protein sequences through a convolutional neural network and combines them into a PPI network for function prediction. Due to the enhancement of protein function prediction by introducing graph information of proteins, more and more methods have started to apply graph neural networks to protein function prediction. DeepFRI [8] structure-based approach treats protein structures as graph neural networks, using structural models from the PDB [9] and SWISS-MODEL [10]. DeepGraphGO [11] utilizes the family and domain information of sequences as the node features of the graph, and then utilizes the graph convolutional network to obtain the structural information of the PPI network. GAT-GO [12] is a method based on the graph attention network, which utilizes a graph attention network to process the predicted structural information and sequence embedding information, which significantly enhances the prediction of protein function.

Currently, protein function prediction methods based on graph neural networks suffer from the following problems:

1. Traditional GNNs (e.g., GCNs) are prone to excessive smoothing problems when stacked in multiple layers, resulting in convergence of node features and difficulty in capturing interactions between distant but functionally related residues in proteins. Secondly, traditional GNNs mostly deal with the internal structure of proteins (e.g., contact maps) and external PPI network topology information in isolation, failing to effectively integrate multilayer biological features (e.g., sequence similarity, structural conservatism, and network community characteristics); moreover, traditional GNNs are insufficiently robust to input noise (e.g., PPI network perturbation) and out-of-distribution data (e.g., unknown proteins), which makes them susceptible to the reliability of the data in practical applications.
2. Just as words and sentences in human language acquire meaning through context and relationships with other linguistic elements, the unique pattern of proteins’ arrangement order makes them have similar contextual information, which integrates multidimensional biological features such as cell type, tissue environment, physiological or pathological state in which the protein is located, and is crucial for understanding its functional specificity. Most of the existing deep learning-based protein function prediction methods do not apply such information to the prediction task, and GNN-based protein function prediction methods are unable to learn the contextual information of proteins, although they can process the information of protein graphs and sequences simultaneously.
3. A recent study [13] has shown that by incorporating the hierarchical structure of the GO graph as additional information into the prediction can enable the annotation model to value the distribution of GO tags, which can benefit the final prediction. However, most GNN-based prediction methods do not extract the information embedded in GO labels, thus failing to effectively capture the interrelationships of GO terms.

Furthermore, some recently proposed methods—such as TALE [14] and DeepGO-SE [15]—despite incorporating advanced sequence modeling or ontology embedding techniques, still suffer from fundamental limitations in their architectural design. Specifically, TALE relies solely on Transformer-based sequence modeling and fails to integrate protein-protein interaction (PPI) networks; while DeepGO-SE leverages ontology axioms through ELEmbeddings, it does not model protein relationship data as graph structures. This lack of bioinformatics processing capabilities for graph structures limits their ability to derive functional insights from network topology, community structure, or interaction context, leaving a critical gap in multi-source functional annotation.

In order to solve these problems mentioned above, we propose a novel protein function prediction method called MAGIN-GO. MAGIN-GO distinguishes itself significantly from previous graph methods based on attention mechanisms or those with only ontology-aware capabilities through two key innovations, effectively addressing the aforementioned gaps.

First, we designed a Graph Convolutional Self-Attention Network (GMSA) module that natively integrates graph convolutions with multi-head self-attention mechanisms. Unlike models that merely apply Transformers to sequences, GMSA operates directly on protein-protein interaction (PPI) graphs, enabling simultaneous capture of long-range dependencies within network topologies and contextual information about proteins.

Second, we move beyond treating Gene Ontology (GO) terms as flat prediction labels or solely for loss calculation. Instead, we incorporate pre-trained GO term structural embeddings as semantic guidance into the core feature fusion process. This allows the model to explicitly align learned protein representations with the hierarchical functional space during prediction, enabling a more nuanced understanding of semantic and hierarchical relationships between labels.

Specifically, the model takes protein sequence information combined with PPI network information as input, and generates node features with stronger expressive ability compared to the traditional GNN model through graph homology network. At the same time, the protein information will also be input to a special Transformer module, which combines the GCN convolutional block with the multi-head self-attention mechanism, and can capture and automatically integrate the long-stroke dependency of the sequence and the protein context information at the same time. The GO embeddings generated by the pre-trained language model Anc2Vec [16] contain the hierarchical structure information of GO annotations, which we finally combine with the protein information obtained from the two graph neural networks and input into a multi-label classifier to classify them in order to output the classification results.

In order to fully validate the feasibility of our approach, we train and evaluate the model on an experimentally annotated dataset, comparing MAGIN-GO to the baseline methods including Naive [17], DeepGOPLUS(improved) [18], DeepGOZero [19], DeepGraphGO [11], TALE [14], SPROF-GO [20], DeepGO-SE [15] and MEGA-GO [21]. The results show that the predictive performance of our method outperforms other state-of-the-art methods, such as DeepGO-SE [15], MEGA-GO [21] and MMSNet [22] which demonstrates that our model adequately captures multi-source information such as protein sequences, protein interactions, protein contexts, and interrelationships of GO annotations, and fuses them sufficiently to successfully improve the prediction accuracy. Meanwhile, our model also exhibits excellent generalization and outstanding interpretability.

Overall, our main contributions can be summarized as follows:

- We employ Graph Isomorphism Network (GIN) to train Protein-Protein Interaction (PPI) graphs integrated with sequence information, enabling the extraction of features that are more expressive compared to those obtained using traditional Graph Neural Networks (GNN).
- We integrate Graph Convolutional Network (GCN) with a multi-head attention mechanism to introduce a specialized transformer module capable of capturing long-range dependencies in sequences while incorporating protein context information.
- We utilize a pre-trained language model to derive embeddings from GO annotations, thereby integrating the hierarchical structure of GO graphs into the prediction network. This enables the annotation model to more effectively capture and emphasize the distribution of GO labels.

## Related work

This study aims to develop an efficient and accurate end-to-end deep-learning protein function prediction method to meet the challenges posed by the increasing amount of high-throughput data. Effective protein function prediction not only solves the high cost problem caused by traditional wet experiments, but also facilitates progress in research areas such as disease prevention and cellular mechanism exploration. In recent years, the development of high-throughput technologies has led to a significant increase in biological data, resulting in the emergence of a large number of protein function prediction methods. These methods are mainly based on mathematical methods for annotation frequency computation and deep learning techniques to enhance the performance of function prediction. In this paper, we discuss eight prediction methods that are closely related to this study: Naive [17], DeepGOPLUS (improved) [18], DeepGOZero [19], DeepGraphGO [11], SPROF-GO [20], TALE [14], DeepGO-SE [15], MEGA-GO [21] and MMSNet [22]. These methods utilize annotation frequency analysis and various deep learning techniques that provide different technical paths for protein function prediction.

First, protein function prediction is mainly based on simple assumptions or basic statistics of techniques such as BLAST [5], k-NN [6], Naive [17], etc. Naive predicts protein function based on the frequency of proteins annotated with specific GO categories. Due to the imbalance of GO category annotations and propagation based on the true path rule, some categories have more annotations than others. Therefore, the same GO category is assigned to all proteins based on the annotation frequency to obtain the prediction results. Here, each query protein is annotated with GO categories and the prediction scores are calculated as a function of each query protein annotation. Although simple and easy to implement, prediction methods based on basic statistical approaches, such as those led by Naive, oversimplify the complexity of biological systems and overlook various properties, such as protein interactions. As a result, this class of methods exhibits limited capability in predicting distant sequences or novel functions.

Since it is difficult to obtain accurate predictions with traditional machine learning methods, more and more deep learning methods based on different neural networks are developed for protein function prediction. These methods are capable of incorporating diverse biological information into the training process of the model, thereby enhancing the model’s sensitivity to biological properties and improving prediction performance. In this paper, we primarily discuss methods that utilize protein maps, protein sequences, and GO terminology information as training features.

Relying on today’s mature gene sequencing technology, protein sequences have become the most versatile deep learning input features. The advantages of DeepGOPLUS [18] are that it does not require the integration of multi-source data and maintains low engineering complexity. DeepGOPLUS predicts the functional annotations of proteins by combining a one-dimensional convolutional neural network (1D-CNN) with the DiamondScore method, and provides a more comprehensive coverage of interactions than structural information. IIn our comparative study, we employ only the 1D-CNN component of DeepGOPLUS and replace the input features from sole one-hot encoding with ESM2 [23], which generates embedding vectors that more effectively capture protein sequence features, resulting in our DeepGOPLUS (improved) method. Although DeepGOPLUS (improved), which relies on sequence information as a single-source feature, eliminates much of the data preprocessing work, using only sequence data fails to capture higher-order structural and dynamic information that also determines certain protein functions. Ignoring this information causes the model to rely heavily on known conserved patterns, resulting in limited predictive capability for novel sequences.

To further utilize the diverse biological network information, more and more prediction methods are starting to use graph neural networks for prediction.

DeepGraphGO [11] introduces graph neural network methods for protein function prediction. DeepGraphGO utilizes InterPro [24] structural domain information as node features in PPI networks. PPI graphs enriched with structural domain information are fed into the graph convolutional network. The graph convolutional network generates functional predictions by integrating structural domain information and network information.

MEGA-GO (Multi-scale Graph Adaptive Neural Network) [21] is a graph neural network method for functional prediction of proteins with different sequence lengths. The method constructs a three-branch Dependent Hierarchical Graph Neural Network (DH-GNN) containing a master branch (dealing with medium-length sequences), a long sequence extractor (Extractor_α_), and a short sequence extractor (Extractor_β_), and realizes the information interaction between the branches through an adaptor block (IAB) to alleviate the oversmoothing problem in the traditional graph neural networks. In the feature processing stage, MEGA-GO utilizes the adaptive feature fusion mechanism (adaAF) to combine solo thermal coding with ESM-1b [25] pre-trained embedding; and dynamically filters key features through adaptive structural attention block (adaSAB) to enhance the structural saliency of proteins with different sequence lengths.

In addition to biological networks, GO ontology-embedded information has gradually become an important source of functional prediction information in recent years. Since GO is often used as a functional annotation label for prediction tasks, incorporating GO term information into the prediction process can enhance the model’s sensitivity to the label and thus improve the prediction performance.

DeepGOZero [19] introduces ontology embedding methods to protein function prediction. Ontology embedding refers to the application of representation learning methods (e.g., word embedding) to learning the embedded representation of an ontology. ELEmbeddings embed ontology semantics into geometric models by representing the categories in the ontology as spheres of arbitrary dimensions and the relations as vectors. DeepGOZero utilizes InterPro structural domains as input and employs a two-layer multilayer perceptron (MLP) module with residual connections, while adopting the ELEmbeddings loss function to learn a representation of GO categories in the embedding space and optimize it through protein function prediction losses.

TALE [14] employs a deep neural network model based on Transformer [26] to predict function and incorporates hierarchical relationships in the GO into the model’s loss function in the model. Deep neural network predictions are combined with sequence similarity-based predictions. The SPROF-GO [20] method utilizes the ProtT5-XL-U50 [27] protein language model to extract protein sequence embeddings and trains a neural network model based on the attention mechanism. The model incorporates the GO hierarchy into the neural network and predicts functions that are consistent with the hierarchical relationships of GO categories. However, as a purely Transformer-based approach, TALE is inherently limited to processing sequential inputs and cannot integrate biological data such as protein-protein interaction (PPI) networks. This restricts its ability to leverage topological features, which are often crucial for inferring protein functions within cellular environments.

DeepGO-SE [15] predicts protein functions through knowledge-enhanced learning and combines them with other information. Specifically, DeepGO-SE (similar to DeepGOZero) projects ESM2 [23] embedding representations into embedding spaces (ELEmbeddings) derived from the axioms of the gene ontology. ELEmbeddings encode the axioms of the ontology using geometrical shapes and geometric relationships, and are optimized jointly with the protein function prediction loss through the ELEmbeddings loss. Although DeepGO-SE excels in semantic embedding, it does not incorporate graph-based structural representations of proteins, such as interaction networks or domain associations. This limits its ability to leverage relational inductive biases prevalent in biological systems—biases that graph-aware models like MAGIN-GO can effectively capture.

AlphaFold2 [28] has revolutionized the field of protein structure prediction. Recently, numerous methods have leveraged AlphaFold2 predictions for functional prediction. Liu et al. proposed MMSNet [22], which integrates AlphaFold2-predicted structural data through a dual-branch architecture. By combining one-dimensional and two-dimensional convolutional neural networks, MMSNet effectively captures both sequence patterns and spatial structural features. However, MMSNet disregards contextual information and cannot fully capture the essential graph structure of biological interactions through convolutional networks alone.

While methods that incorporate biological network or GO term information into functional prediction are superior in performance to methods that make predictions based on sequence information alone, few methods incorporate both biological network information and GO term structure information into the model for prediction. At the same time, these methods ignore the contextual and long-range information of protein sequences, and the loss of this information can lead to the failure of the model to capture the complete information of functional and structural domains, thus affecting the prediction performance of the model.

In conclusion, the above eight methods provide different solutions in different areas of deep learning and have made significant progress in the areas of gene and protein function prediction, respectively. The above methods provide important theoretical and practical references for the further development and improvement of gene function prediction techniques in this study, and our method addresses the shortcomings mentioned in the above eight methods and designs a model with better prediction performance.

## Materials and methods

### Datasets

This study is a computational analysis of publicly available data and does not require any specific permits or ethical approvals.

#### UniProtKB/Swiss-Prot dataset.

In this experiment, we employ the same dataset as DeepGO-SE [15], which is available at (https://github.com/bio-ontology-research-group/deepgo2). The dataset was derived from the manually curated and reviewed protein dataset in the UniProtKB/Swiss-Prot Knowledgebase [2] version 2021-04 released on September 29, 2021, and extracted from the experimentally acquired data, i.e., the evidence codes EXP, IDA, IPI, IMP, IGI, IEP, TAS, IC, HTP, HDA, HMP, HGI, and HEP, HGI and HEP proteins. The dataset contains a total of 77,647 reviewed and manually annotated proteins. In order to exclude as much as possible the influence of sequence similarity of proteins on the prediction results of the model, this dataset was divided into datasets based on the similarity match with the maximum expected value (e - value) score of 0.001, and the similarity between two pairs of proteins was computed by using Diamond (v.2.0.9) [29], and sequences with certain similarity were grouped together, and then these groups were classified into the training, validation, and test sets (see Table 1).

**Table 1:** Summary of the UniProtKB/Swiss-Prot dataset.

Ontology	GO Terms	Proteins	Training	Validation	Testing
MF	6,851	43,279	38,533	1,901	2,845
BP	21,356	58,729	52,584	2,870	3,275
CC	2,829	59,257	52,072	2,964	4,221

Protein function annotation is described by GO terms [4]. GO employs a directed acyclic graph (DAG) to model the relationships between GO terms. Nodes represent GO terms, and the edges of the DAG represent relationships between terms. GO provides three separate directed acyclic graphs (DAGs) for each of the three ontologies (BP, CC and MF), with the shallow terms representing broad, abstract semantics, and the deeper terms representing concrete, precise semantics. Associating a protein with a specific term means that the protein is also associated with all ancestral terms in the term hierarchy. Thus, if a term is annotated to a protein, its ancestor terms are automatically annotated to the same protein by referring to the GO hierarchy and the True Path [30]. For each protein, the specific GO terms provided in the UniProt functional annotation file were first collected. Then, the parent and ancestor terms of these terms in the GO DAG were also collected. Based on this dataset, we empoly the GO released on November 16, 2021 as the functional annotation label. We train and evaluate the models separately for each sub-ontology of GO.

#### neXtProt dataset.

To further evaluate the effectiveness of our model, we employ a prediction dataset sourced from the neXtProt database, which contains human proteins that have been manually annotated but whose biological functions remain undetermined. neXtProt serves as an integrated platform for the standardization and consolidation of information related to human proteins, offering users enhanced search capabilities based on semantic technologies. The database compiles free-text descriptions derived from scientific literature, standardized enzyme annotations from UniProtKB/Swiss-Prot, pathway information from KEGG and Reactome, as well as GO terms covering MF and BP from diverse resources. These annotations may be either manually curated by experts or generated through automated procedures, and they can be derived from experimental studies or computational analyses.

In this study, we collected and integrated data from 113 peer-reviewed publications along with other relevant resources to perform functional predictions for 239 uncharacterized human proteins. In total, 659 specific GO annotations were assigned to these proteins, including 69 molecular function annotations across 53 proteins and 590 biological process annotations distributed among 225 proteins. Approximately one-third of the proteins (38%) were assigned only a single functional annotation, and among these, 85% corresponded to BP terms. Moreover, the majority of the predicted functions (78%) were supported by only a single line of evidence.

### Input features

#### protein embeddings.

To obtain residue-level protein sequence embeddings, we obtain embeddings through the protein language model ESM2 [23], a large-scale Transformer architecture trained on protein sequences. It is based on more than 250 million protein sequences and trained in conjunction with the UniRef UR50/50 database [31]. ESM embeddings have been successfully applied to several protein engineering tasks, such as directed-guided evolution. The second version of ESM (ESM2) has been improved to utilize more parameters for pre-training. Compared to the previous version, ESM-1b [25], ESM2 is able to learn superior representations, and these learned representations can also used for protein tertiary structure prediction. We utilize the pre-trained ESM2 model with 3 billion parameters to generate a dataset that characterizes proteins. Specifically, for a protein *p* with *M* amino acids, we utilize ESM2 to obtain the embedding of each amino acid, forming a matrix $B\in {\mathbb{R}}^{M\times d_{1}}$, where *d*_1_ is the amino acid embedding dimension and its size is fixed to 2560. Following established practices in the literature, we apply the same average pooling strategy to aggregate the amino acid features, yielding the protein embedding $f(p)=\text{mean}(B)\in {\mathbb{R}}^{1\times d_{1}}$. We selected mean pooling over alternative approaches (such as max pooling or attention-based pooling) because it provides stable and generalizable representations across entire sequences. This method is widely adopted in protein function prediction tasks and serves as a proven strategy for capturing holistic protein characteristics [15].

#### Protein-protein interaction network.

PPI networks construct dynamic topologies with proteins as nodes and interactions as edges by integrating experimental validation (e.g., yeast two-hybrid, Co-IP) and computational prediction data (e.g., STRING database). The PPI network analysis tool identifies key targets through topological metrics, such as degree distributions and clustering coefficients, and provides a multi-omics integration strategy for the construction of disease-specific networks and drug development (e.g., disrupting disease-causing protein interactions) provides a multi-omics integration strategy. Therefore, incorporating multiple proteins and their interactions in protein prediction experiments can help to discover the molecular functions of proteins. Our experiments are also conducted using the DeepGO-SE dataset, which includes an interaction network containing a total of 1.24 million interaction pairs. We utilize all the protein interaction graphs in this dataset and utilize the DGL [32] library to process them and train graph neural networks.

#### GO term embedding.

For gene ontology embeddings, we apply the pre-trained model Anc2vec [16] to generate compact GO term embeddings. Anc2vec is a neural network-based framework that leverages three structural characteristics of Gene Ontology (GO) terms: ontological uniqueness, ancestry hierarchy, and affiliation with sub-ontologies. Experiments show that Anc2vec is able to capture these features efficiently and outperforms existing embedding methods, especially on datasets annotated with GO terms. More specifically, each GO term *G*_*i*_ is embedded into a *d*_0_ dimensional label representation vector, where *d*_0_ is a predefined number of hidden dimensions. Here, we set *d*_0_ to 128.

### Model and implementation

**Fig 1:**
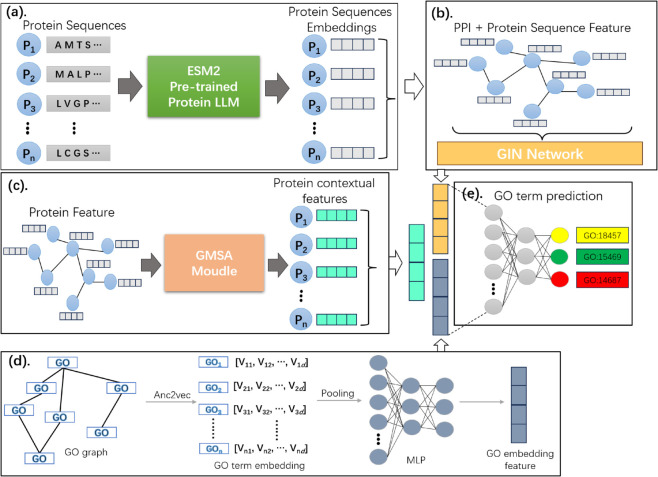
The framework of the MAGIN-GO. (a) The GIN module learns local topological patterns in the PPI network to generate graph-enhanced features; (b) Sequence embedding features are fused with the PPI network to construct protein representations; (c) The GMSA module captures long-range dependencies and global contextual interactions within and between sequences, complementing the local network features from GIN; (d) Pre-trained GO term embeddings are fused with the dual-module output features; (e) The fused features enable protein functional annotation prediction through a residual classifier.

MAGIN-GO is a multi-label classification model designed to predict multiple GO term functions of proteins. As shown in Fig 1, this framework integrates three synergistic components: graph isomorphism networks, GCN multi-head self-attention modules, and Anc2vec embeddings.

The MAGIN-GO framework is a highly synergistic system where interconnected components complement each other’s strengths. The GIN and GMSA modules process the same PPI graphs and sequence features in parallel, yet employ distinct inductive bias strategies: GIN focuses on local fine-grained graph structures through robust homogeneity-aware aggregation, while GMSA leverages graph-compatible self-attention mechanisms to model global long-range dependencies across the entire network. Features from both graph networks are fused with pre-trained GO embeddings. Here, GO embeddings serve as semantic guides, directing the fused representations toward a semantic space that captures hierarchical relationships among GO functional terms.

Protein sequences from ESM2 embed node features into the initial PPI graph, while averaged GO term embeddings provide ontology context. These multi-source features are integrated via a multi-label classifier to generate functional predictions.

#### Graph isomorphism network (GIN).

At this stage, to integrate PPI with sequence features, we represent the PPI network as an adjacency matrix and utilize the protein sequence features derived from ESM2 as the node attributes of the graph. Subsequently, we employ a graph isomorphism network (GIN) to propagate these features among residues with analogous structures [33]. The GIN is theoretically capable of proving to satisfy the conditions related to the graph isomorphism test by the generalization of the Weisfeiler-Lehman (WL) test, and theoretically possesses the maximum discriminative power among traditional graph neural networks.

In this experiment, we design a GIN architecture consisting of two hierarchical GIN convolutional layers, and the hidden layer dimension of each GIN convolutional layer is set to 1024 and the dropout is set to 0.3. Formally, we are given the PPI graph $g_{t}=(V_{t},X_{t})$,where $X_{t}\in {\mathbb{R}}^{m\times d_{1}}$ is the feature matrix of the PPI graph,The column vectors of its feature matrix come free of the protein sequence features generated by ESM2. *m* denotes the number of nodes of the PPI graph. The core aggregation function of the two GIN convolutional layers can be defined as Eq (1):

$$x_{v}^{\left(k\right)}={MLP}^{\left(k\right)}\left(\left(1+\epsilon^{\left(k\right)}\right)\cdot x_{v}^{\left(k-1\right)}+\max_{u\in 𝒩(v)}x_{u}^{\left(k-1\right)}\right)$$ (1)

In this context, $x_{v}^{\left(k\right)}$ represents the representation of the protein after the *k*-th layer of the GIN convolution module. 𝒩(v) denotes the set of neighboring nodes of node *v*, and *ε* is a scalar parameter that can be learned to enhance the self-connectivity. The term max indicates that the GIN convolution layer employs a maximum aggregation strategy. After obtaining the output of the first layer, we concatenate the output of the first layer with the features of the cross-layer adjacency matrix to form the enhanced features. These enhanced features are then fed into the second GIN convolutional layer to obtain the corresponding output $X_{GIN}\in {\mathbb{R}}^{m\times d_{2}}$ of the GIN module.

#### GCN-based multi-head self-attention (GMSA).

**Fig 2:**
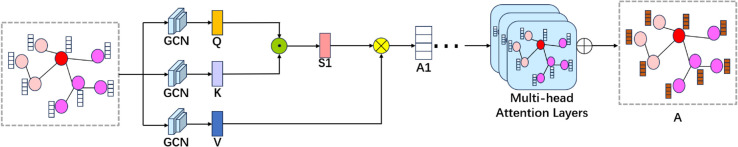
The structure of the GMSA. The structure of the GMSA module involves processing the inputs through a GCN to generate the query vector (*Q*), key vector (*K*), and value vector (*V*). Protein context information is then captured using a multi-head self-attention mechanism.

Since combining the attention mechanism into the GIN module alone will dilute the contextual information, GMSA will extract the information independently of GIN considering the integrated feature information. As shown in Fig 2, in the GMSA module, we integrate a graph convolutional network with a multi-head self-attention mechanism by incorporating a Transformer [26] architecture. This module leverages the node features of the PPI graph to capture long-range dependencies within protein sequences. The primary objective is to learn features that are distinct from those obtained using the GIN. Additionally, the unique architecture of the Transformer enables the effective capture of contextual information related to proteins. Specifically, the GMSA model contains two cascaded graph convolution layers, where the input feature $X_{t}\in {\mathbb{R}}^{m\times d_{1}}$ with the PPI graph *g*_*t*_ is fed into the first level graph convolution layer GCN1. The computation formula of GCN is shown in Eq (2) [34]:

$$H^{\left(l+1\right)}=\text{ReLU}\left({\tilde{D}}^{-0.5}\tilde{A}{\tilde{D}}^{-0.5}H^{\left(l\right)}W_{g}^{\left(l\right)}\right)$$ (2)

Where $H^{\left(l\right)}\in {\mathbb{R}}^{m\times d_{l}}$ denotes the node feature matrix of layer *l* and $H^{\left(0\right)}=X_{t}$. $W_{g}^{\left(l\right)}\in {\mathbb{R}}^{d_{l}\times d_{l+1}}$ is the weight matrix of layer *l*. $\tilde{A}=A\;+\;I$ is the adjacency matrix of the graph *g* with the addition of self-connections, and $\tilde{D}$ is the degree matrix of $\tilde{A}$, where ${\tilde{D}}_{ii}=\sum_{j}{\tilde{A}}_{ij}$. The ReLU function introduces non-linearity, and the symmetric normalization ${\tilde{D}}^{-1/2}\tilde{A}{\tilde{D}}^{-1/2}$ enables effective neighborhood aggregation in the GCN layer. The Query (*Q*), Key (*K*) and Value (*V*) triples are generated by the first level graph convolutional layer GCN1. The *Q*, *K* and *V* triples are generated in Eq (3):

$$\text{ReLU}\left(\text{GCN1}\left(H^{\left(0\right)}\right)\right)$$ (3)

In our experiments, the GMSA module employs *h* = 8 parallel attention heads. The *Q*, *K* and *V* vectors of each attention head are projected to dimension *D* = *d*_2_/*h* = 128.

Split *Q*, *K*, and *V* into a specified number of heads and calculate the attention weights by calculating the similarity between the query vectors and the key vectors as shown in Eq (4), and then the attention weights are weighted on the value vectors to obtain $\Psi_{i}\in {\mathbb{R}}^{m\times D}$.

$$\Psi_{i}=\text{softmax}\left(\frac{Q_{i}K_{i}^{⊤}}{\sqrt{D}}\right)V_{i}$$ (4)

Where *Q*_*i*_, *K*_*i*_ represent the query vector and key vector of the *i*-th head, respectively, and their dot product is divided by a scaling factor. Then, softmax is applied to compute the attention weight and multiplied with $V_{i}$ to obtain the weighted features of the current head. The weighted features computed at each node are spliced with each other to obtain $A\in {\mathbb{R}}^{m\times d_{2}}$ as Eq (5):

$$A=[\Psi_{1},\Psi_{2},\ldots ,\Psi_{H}]$$ (5)

This multi-head output *A* is passed through the second level of graph convolution GCN2 to integrate the neighborhood information and output the contextual features:

$$X_{GMSA}=\text{LayerNorm}\left(\text{GCN2}\left(A\right)\right)\in {\mathbb{R}}^{m\times d_{2}}$$ (6)

#### GO term prediction module.

In addition to the features generated by GIN and GMSA, we further incorporate embeddings of GO terms as GO aspect features. The label representation vectors $G\in {\mathbb{R}}^{L\times d_{0}}$ of GO terms are first average pooled to aggregate the labeled features, and the pooled label vectors are inputted to an MLP to project the label vectors to the protein feature equivalent dimensions and fused:

$$X_{GO}=\text{Dropout}\left(\text{LayerNorm}\left(\text{ReLU}\left(\text{MLP}\left(G\right)\right)\right)\right)$$ (7)

$$X_{all}=X_{GIN}+X_{GMSA}+X_{GO}$$ (8)

Where $X_{GIN}\in {\mathbb{R}}^{m\times d_{2}}$, $X_{GMSA}\in {\mathbb{R}}^{m\times d_{2}}$, and $X_{GO}\in {\mathbb{R}}^{m\times d_{2}}$ are dimensionally compatible feature matrices. Specifically, we ensure dimensional consistency by configuring the hidden layer output dimension of the multilayer perceptron in formula (7) to *d*_2_ = 1024, aligning it with the output dimensions of the GIN and GMSA modules. This dimensional alignment enables direct feature fusion through summation, preserving computational efficiency while fully retaining the structural information from all three sources.

Additionally, we explored the following approaches for feature fusion. Concatenation: Concatenating the three features column-wise slightly increased the number of parameters but did not improve performance. Label-wise Attention: Calculated attention over protein features using GO embeddings as queries. However, this method incurred high computational overhead and exhibited unstable training behavior. Weighted Sum: Learned weighted combinations of different ontology embeddings to compute each feature’s contribution. While this approach increased model complexity, it failed to yield significant gains. Ultimately, we selected element-wise addition due to its simplicity, stability, and efficiency.

Then, we propose and utilize an improved residual network module that combines the idea of residual learning with the classic multi-layer perceptron architecture, offering excellent expressive power and stability. The module primarily consists of an input layer, a residual connection, and an output layer. In the definition of the input layer, our input vector is $X_{all}\in {\mathbb{R}}^{m\times d_{2}}$, and the input data undergoes a linear transformation through a fully connected layer, where $W_{1}\in {\mathbb{R}}^{d_{2}\times d_{1}}$ and $b_{1}\in {\mathbb{R}}^{d_{2}}$ represent the weight matrix and bias term, respectively. Next, the ReLU activation function is applied to introduce nonlinear features, and LayerNorm is used to normalize the activation values. After normalization, a Dropout operation is applied to prevent overfitting:

$$\tilde{X}=\text{Dropout}\left(\text{LayerNorm}\left(\text{ReLU}\left(W_{1}X_{all}+b_{1}\right)\right)\right)$$ (9)

In residual connections, we fuse the original information through simple element addition, and the structure allows the network to learn the identity function more easily during training, thereby improving the trainability of the network.

$$X_{res}=\tilde{X}+X_{all}$$ (10)

The final tag-specific logits are generated by a linear layer acting on the residual-connected features $X_{res}$. Although GO embeddings are averaged at the ontology level as mentioned earlier, the final classifier remains responsible for learning unique, fine-grained associations between the fused protein–GO features and each specific GO tag. This design strikes an effective balance by incorporating a universal ontology structure while preserving the ability to distinguish specific tags.

## Experiment and results

### Model training

During the training phase, the Adam optimizer [35] is employed to train the proposed MAGIN-GO model. The learning rate is set at 0.001, the batch size is configured to 37, and the training is conducted over 256 iterations. The method is implemented based on pytorch and DGL libraries and the pre-trained Anc2vec is implemented based on tensorflow geometry library. To prevent unnecessary training when no performance improvement is observed, an early stopping strategy is employed. Training is conducted on an NVIDIA GeForce RTX 4070 12G GPU.

### Evaluation metrics

In this study, we employ four distinct evaluation metrics to comprehensively assess the performance of our model from multiple perspectives. These metrics include the maximum F-value (Fmax) [36], Smin [37], the area under the precision-recall curve (AUPR) [38], and one class-centric AUC [39]. The Fmax metric represents the maximum F-value computed under all the prediction thresholds (in the range of [0,1] and with a step size of 0.01). Smin denotes the semantic distance between the predicted annotations and the true annotations, taking into account the content information of each feature. AUPR summarizes the precision-recall curves by approximating the weighted average of the precision at each threshold by the trapezoidal rule, and is used to calculate the area under the precision-recall curve to evaluate the performance of the model at different prediction thresholds. AUC is a category-centric measure that The AUC ROC for each category is calculated and averaged. Among these evaluation metrics, the smaller the value of Smin the better the model performance, and Fmax, AUPR, and AUC all have larger values the better the model performance.

### Comparison of MAGIN-GO with other methods

To evaluate the performance of MAGIN-GO, the UniProtKB/Swiss-Prot dataset is divided into training, validation and test sets. We focus on three domains of gene ontology: molecular function (MF), biological process (BP) and cellular component (CC). We compare the prediction results of MAGIN-GO on the test set with the mainstream protein function prediction models in recent years, which include Naive [17], DeepGOPLUS(improved) [18], DeepGOZero [19], DeepGraphGO [11], TALE [14], SPROF-GO [20], DeepGO-SE [15], MEGA-GO [21] and MMSNet [22].

Among the four metrics, MAGIN-GO consistently outperforms these methods. The Fmax values of MAGIN-GO for the three Gene Ontology (GO) domains MF, BP, and CC are 0.568, 0.458, and 0.752, respectively. The AUPRC values are 0.569, 0.434, and 0.754, respectively. The Smin values are 11.279, 37.709, and 8.079, respectively. The AUC values are 0.896, 0.897, and 0.940, respectively. Specific results are summarized in Tables 2 and 3. We found that MAGIN-GO demonstrates particularly outstanding predictive performance in the MF domain, primarily due to the hierarchical and semantically related characteristics of labels in this domain aligning highly well with the GO structural information extracted by Anc2Vec.

**Table 2:** Experimental results on UniProtKB/Swiss-Prot data - Part 1 (mean ± std).

Method	AUPR (↑)			Fmax (↑)		
	MF	BP	CC	MF	BP	CC
Naive	0.180 ± 0.000	0.195 ± 0.000	0.490 ± 0.000	0.321 ± 0.000	0.294 ± 0.000	0.620 ± 0.000
DeepGOPLUS(improved)	0.433 ± 0.017	0.318 ± 0.019	0.694 ± 0.021	0.449 ± 0.023	0.365 ± 0.024	0.679 ± 0.019
DeepGOZero	0.444 ± 0.026	0.284 ± 0.028	0.587 ± 0.030	0.448 ± 0.032	0.343 ± 0.034	0.625 ± 0.027
DeepGraphGo	0.357 ± 0.036	0.303 ± 0.038	0.666 ± 0.040	0.416 ± 0.042	0.354 ± 0.044	0.667 ± 0.037
SPROF-GO	0.485 ± 0.021	0.332 ± 0.023	0.690 ± 0.025	0.515 ± 0.027	0.391 ± 0.029	0.693 ± 0.022
MEGA-GO	0.505 ± 0.011	0.402 ± 0.013	0.715 ± 0.015	0.525 ± 0.017	0.401 ± 0.019	0.690 ± 0.012
DeepGO-SE	0.539 ± 0.031	0.398 ± 0.033	0.723 ± 0.035	0.541 ± 0.037	0.428 ± 0.039	0.713 ± 0.032
TALE	0.472 ± 0.023	0.328 ± 0.024	0.679 ± 0.025	0.504 ± 0.026	0.378 ± 0.027	0.681 ± 0.028
MMSNet	0.532 ± 0.019	0.393 ± 0.020	0.711 ± 0.021	0.522 ± 0.022	0.423 ± 0.018	0.724 ± 0.019
**MAGIN-GO**	**0.569 ± 0.013**	**0.434 ± 0.014**	**0.754 ± 0.015**	**0.568 ± 0.016**	**0.458 ± 0.017**	**0.752 ± 0.018**

Table notes: Arrows (↑) indicate higher is better; (↓) indicate lower is better.

**Table 3:** Experimental results on UniProtKB/Swiss-Prot data - Part 2 (mean ± std).

Method	Smin (↓)			AUC (↑)		
	MF	BP	CC	MF	BP	CC
Naive	14.568 ± 0.000	43.934 ± 0.000	11.879 ± 0.000	0.500 ± 0.000	0.500 ± 0.000	0.500 ± 0.000
DeepGOPLUS(improved)	13.097 ± 0.025	41.609 ± 0.035	10.477 ± 0.030	0.818 ± 0.012	0.813 ± 0.015	0.854 ± 0.018
DeepGOZero	12.722 ± 0.038	42.857 ± 0.042	11.700 ± 0.040	0.881 ± 0.022	0.643 ± 0.025	0.599 ± 0.028
DeepGraphGo	14.077 ± 0.045	42.100 ± 0.043	10.020 ± 0.038	0.673 ± 0.032	0.736 ± 0.034	0.814 ± 0.036
SPROF-GO	12.231 ± 0.018	40.523 ± 0.028	10.447 ± 0.022	0.786 ± 0.020	0.802 ± 0.024	0.878 ± 0.026
MEGA-GO	12.192 ± 0.015	40.053 ± 0.025	10.262 ± 0.020	0.867 ± 0.008	0.839 ± 0.010	0.891 ± 0.014
DeepGO-SE	11.942 ± 0.028	39.687 ± 0.032	9.713 ± 0.026	0.872 ± 0.016	0.855 ± 0.018	0.910 ± 0.020
TALE	12.503 ± 0.035	40.829 ± 0.038	10.812 ± 0.033	0.840 ± 0.030	0.773 ± 0.028	0.847 ± 0.032
MMSNet	12.043 ± 0.022	39.605 ± 0.030	9.183 ± 0.024	0.853 ± 0.014	0.805 ± 0.016	0.873 ± 0.012
**MAGIN-GO**	**11.297 ± 0.008**	**37.709 ± 0.012**	**8.079 ± 0.010**	**0.896 ± 0.005**	**0.897 ± 0.006**	**0.940 ± 0.007**

Table notes: Arrows (↑) indicate higher is better; (↓) indicate lower is better.

Among existing methods, DeepGraphGO and MEGA-GO, though built upon graph neural networks, fail to integrate the semantic and structural information of GO terms. Consequently, they lag behind MAGIN-GO in the MF prediction task. Although DeepGO-SE incorporates GO logical axioms via ELEmbeddings and performs well on certain dependency hierarchy tasks, MAGIN-GO achieves superior overall performance across most metrics by integrating PPI network structure with GO hierarchical information. It is noteworthy that while the recently proposed MMSNet innovatively integrates structural information predicted by AlphaFold2, its modeling remains confined to monomeric protein structures and fails to incorporate the system-level insights provided by PPI networks. Experimental results demonstrate that MAGIN-GO outperforms MMSNet across all three ontology functions. Furthermore, under identical experimental conditions, MAGIN-GO requires significantly less training time than DeepGO-SE, TALE, and SPROF-GO, achieving a balance between accuracy and efficiency. The efficiency comparison of DeepGO-SE, TALE, and MAGIN-GO is shown in Table 4:

**Table 4:** Computational efficiency comparison.

Method	GPU hours	Training Time(S)	Parameter Count(M)
DeepGO-SE	10.20	36886.91	≈129.0
TALE	7.32	26352.81	≈76.8
MAGIN-GO	4.59	16533.09	≈59.1

From a model architecture perspective, MAGIN-GO’s advantage stems from the synergistic design of its dual graph neural network architecture. The GIN module, leveraging its high discriminative power for graph isomorphisms, effectively captures local topological patterns within protein interaction networks. Meanwhile, the GMSA module combines multi-head self-attention mechanisms with graph convolutions to form a specialized Transformer capable of processing graph data, thereby modeling long-range dependencies in protein sequences and structures. This design addresses the shortcomings of traditional GNNs in modeling global dependencies while overcoming the limitations of conventional Transformers in handling graph-structured inputs. For instance, the TALE method, also based on Transformers, suffers from an inherent flaw in perceiving topological features due to its inability to integrate PPI network graph structure information. Consequently, its predictive performance falls significantly below that of MAGIN-GO.

### Ablation study

To investigate the specific impact of each component of MAGIN-GO on its performance, we conduct an ablation experiment that introduces three variants, each retaining the hyperparameter configuration from the previous section of the experiment, while removing different modules and testing them separately. The specific results of MAGIN-GO and its three variants on the dataset are shown in Table 5.

**Table 5:** Ablation experiment results on UniProtKB/Swiss-Prot data.

Method	AUPR(↑)			Fmax(↑)			Smin(↓)			AUC(↑)		
	MF	BP	CC	MF	BP	CC	MF	BP	CC	MF	BP	CC
Without Anc2vec	0.531	0.395	0.741	0.536	0.428	0.730	12.098	39.096	8.874	0.855	0.878	0.922
Without GMSA	0.543	0.416	0.741	0.544	0.436	0.738	11.715	38.230	8.540	0.866	0.877	0.930
Without GIN	0.517	0.408	0.742	0.521	0.436	0.732	12.167	38.848	8.795	0.874	0.884	0.919
**MAGIN-GO**	**0.569**	**0.434**	**0.754**	**0.568**	**0.458**	**0.752**	**11.297**	**37.709**	**8.079**	**0.896**	**0.897**	**0.940**

Table notes: Arrows (↑) indicate higher is better; (↓) indicate lower is better.

The results of the ablation experiments show that deleting any of the components leads to varying degrees of loss in model performance, which is a good proof that all the modules of our model are valid and that MAGIN-GO integrates to more information compared to the other three variants. Under the same experimental setup, the variant with the GIN module removed exhibits the poorest performance among all variants. This observation demonstrates that the introduction of the GIN network to learn enhanced graph features is both important and beneficial. Since protein-related data is typically sparse, the aggregation function of GIN, based on graph isomorphism tests, is capable of learning structural information that cannot be captured by the GCN, thereby enhancing the graph features.

While keeping GIN unchanged, the performance of the two variants with Anc2vec and GMSA removed, respectively, is similar. However, the performance of the variant with Anc2vec removed is generally lower than that of the variant with GMSA removed. This result demonstrates the importance and benefit of learning structural representations of GO terms and optimizing the mapping between protein features and semantic representations. This finding is reflected in the gap in Smin, which is significantly better for the variant that retains the Anc2vec embedding method than the one that deletes Anc2vec, since Smin is defined according to the GO hierarchy. Notably, removing the GMSA module significantly impaired performance for long-sequence proteins (>500 amino acids) compared to short-sequence proteins. Specifically, for MF, the Fmax score decreased by 7.3% for long sequences, whereas short sequences (<300 amino acids) showed only a 3.1% decline. This result clearly demonstrates that GMSA’s attention mechanism is crucial for capturing long-range dependencies in extended protein sequences. Conversely, removing the GIN module had a relatively uniform impact on performance across sequences of different lengths, but particularly affected the prediction of functions dependent on local structural motifs, which require precise topological information. This complementary behavior validates the rationale behind our dual-graph architecture design, proving that the two modules play irreplaceable roles in processing different types of protein information.

As shown in the results of Guan et al. (2024) [40], incorporating protein context information in the prediction task enhances the input features required for the prediction model and thus enhances the downstream task. This ablation experiment further confirms the necessity of introducing dual graph neural network architecture and GO structure information to improve prediction accuracy.

### Ablation study of GO embedding methods

Building upon existing ablation studies, we designed ablation experiments tailored to multiple GO term embedding methods to validate the superiority of our chosen Anc2vec approach. Onto2vec [41] employs a word2vec-like embedding method to process annotated data, learning representations based on the co-occurrence patterns of GO terms within protein annotations. node2vec [42] provides a general-purpose graph embedding method, which we applied directly to the GO graph structure without adapting it for ontology-specific characteristics. We maintained the remaining MAGIN-GO architecture unchanged for this experiment, replacing only the GO embedding method.

As shown in Table 6, although all embedding methods outperformed the baseline model without GO embedding, Anc2Vec achieved the best overall performance across most metrics. This advantage was particularly pronounced in MF, where hierarchical relationships among functional terms are most critical. All embedding methods outperformed the baseline model without GO embedding, Anc2Vec achieved the best overall performance across most metrics. This advantage was particularly pronounced in MF, where hierarchical relationships among functional terms are most critical. As a general-purpose graph embedding method, node2vec lacks ontology-specific semantic awareness, resulting in comprehensive underperformance relative to Anc2Vec across this task. Onto2vec demonstrates strong performance on the Fmax metric by leveraging term co-occurrence patterns in protein annotations, yet still lags behind Anc2Vec on all other evaluation metrics.

**Table 6:** Ablation experiment results on UniProtKB/Swiss-Prot data.

Method	AUPR(↑)			Fmax(↑)			Smin(↓)			AUC(↑)		
	MF	BP	CC	MF	BP	CC	MF	BP	CC	MF	BP	CC
Without GO Embeddings	0.531	0.395	0.741	0.536	0.428	0.730	12.098	39.096	8.874	0.855	0.878	0.922
Onto2vec	0.558	0.426	0.743	**0.571**	**0.460**	**0.756**	11.503	38.147	8.256	0.889	0.890	0.932
node2vec	0.550	0.419	0.735	0.551	0.442	0.745	11.928	38.744	8.475	0.881	0.884	0.927
**MAGIN-GO**	**0.569**	**0.434**	**0.754**	0.568	0.458	0.752	**11.297**	**37.709**	**8.079**	**0.896**	**0.897**	**0.940**

Table notes: Arrows (↑) indicate higher is better; (↓) indicate lower is better.

### Validation of the effectiveness of GIN

To verify the effectiveness of the GIN in protein function prediction, we replace the GIN module in our model with various alternative variants. Experimental results demonstrate that our model attains optimal performance across all variants, as illustrated in Table 7. When extracting protein features from the protein interaction network, we keep the GMSA and Anc2vec embedding modules unchanged and replace GINConv in the original model with three different graph convolution layers, GraphConv [34], GATConv [43], and SAGEConv [44], respectively. The experimental results indicate that the three variants of the aggregation function exhibit comparable performance. However, GINConv outperforms these variants, achieving superior results. Notably, the performance enhancement of GINConv is more pronounced in the prediction of MF compared to the relatively modest improvements observed in BP and CC predictions. Taking GCNConv as an example, GINConv improves 8.19% on Fmax, 7.04% on Smin, 8.59% on AUPR, and 4.55% on AUC over GCNConv. This proves that GIN is significantly better at capturing molecular level features than the rest of the traditional graph neural networks. As stated by Jiang et al [45]. The accuracy of Graph Isomorphism Network (GIN) is significantly higher than that of traditional graph neural networks in predicting molecular properties. This is because GIN is more capable of capturing structural differences during the graph embedding generation stage, thereby enhancing the performance of Molecular Function Ontology (MFO) for downstream prediction tasks.

**Table 7:** Effectiveness of GIN experiment results on UniProtKB/Swiss-Prot data.

Method	AUPR(↑)			Fmax(↑)			Smin(↓)			AUC(↑)		
	MF	BP	CC	MF	BP	CC	MF	BP	CC	MF	BP	CC
GCN	0.524	0.409	0.737	0.525	0.435	0.737	12.153	39.112	8.592	0.857	0.882	0.928
GAT	0.534	0.416	0.733	0.534	0.443	0.735	11.986	38.773	8.576	0.882	0.881	0.931
GraphSAGE	0.543	0.422	0.728	0.546	0.447	0.734	11.776	38.402	8.544	0.873	0.879	0.930
**MAGIN-GO**	**0.569**	**0.434**	**0.754**	**0.568**	**0.458**	**0.752**	**11.297**	**37.709**	**8.079**	**0.896**	**0.897**	**0.940**

Table notes: Arrows (↑) indicate higher is better; (↓) indicate lower is better.

### neXtProt manual prediction dataset evaluation

**Fig 3:**
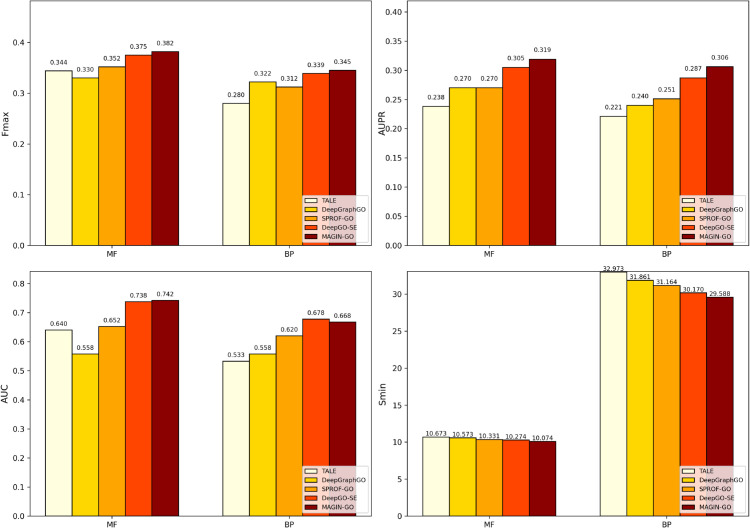
Evaluation metrics comparison among different models.

We utilize the neXtProt dataset to further compare the performance of our method and the baseline method. The neXtProt dataset contains a large number of manually annotated uncharacterized human proteins, enabling the dataset to fully evaluate the model’s predictive capability on real uncharacterized proteins rather than proteins with existing annotations, thereby better reflecting the model’s generalizability and ability to discover new functions. As shown in Fig 3, we find that for molecular function, MAGIN-GO outperformed other baseline methods across all four metrics. It is worth noting that for biological processes, our method shows a decrease in AUC compared to DeepGO-SE [15]. This is because the ELEmbeddings [15] embedding method in DeepGO-SE combines GO logical axioms, which can express more refined GO term information compared to Anc2vec embedding, making DeepGO-SE perform best in term-centric AUC. However, MAGIN-GO outperforms other baseline methods on the other three metrics, and it is more lightweight than DeepGO-SE and achieves superior prediction performance in significantly less training time.

### Testing on targets of the third CAFA challenge (CAFA3)

To assess the robustness of our method, we retrained and tested multiple approaches, including MAGIN-GO, using the Critical Assessment of Protein Function Annotation (CAFA3) dataset. The CAFA3 dataset contains extensive data from TrEMBL, providing a comprehensive test of a model’s noise tolerance and robustness. In this experiment, we primarily selected proteins from the yeast and human species within the CAFA3 dataset and divided these data into training and testing sets, as shown in Table 8. Subsequently, we selected four prominent prediction methods from the baseline approaches and compared them with MAGIN-GO.

**Table 8:** Summary of the CAFA3 dataset.

Ontology	GO Terms	Proteins	Training	Testing
MF	3,516	5,657	4,475	1,182
BP	5,050	8,275	7,863	412
CC	3,814	5,688	4,576	1,112

Table 9 presents the prediction performance comparison of the five methods on the CAFA3 dataset, covering four metrics: Fmax, Smin, AUPR, and AUC. Our method outperforms DeepGOSE and DeepGOPLUS (improved) but falls short of MEGA-GO and MMSNet. This discrepancy likely stems from mismatches between MAGIN-GO’s model architecture and the evaluation scenario of CAFA3. Specifically, the feature distribution learned from training on the UniProtKB/Swiss-Prot dataset differs significantly from the CAFA3 test set, leading to reduced generalization. Notably, the CAFA3 evaluation protocol emphasizes predicting novel proteins, while MAGIN-GO’s dual-graph neural network architecture relies on protein-protein interaction networks, limiting its performance for target proteins with incomplete annotations or sparse network connectivity. Compared to MEGA-GO, which is specifically optimized for different sequence lengths, and MMSNet, which integrates AlphaFold2 structural predictions, MAGIN-GO fails to fully leverage the complementary advantages of multi-source information when handling the heterogeneous data distribution characteristic of CAFA3. This is particularly evident in its suboptimal performance on the Smin metric within the BP domain, reflecting the model’s inadequacy in modeling functional semantic distance.

**Table 9:** Experiment results on CAFA3 data.

Method	AUPR(↑)			Fmax(↑)			Smin(↓)			AUC(↑)		
	MF	BP	CC	MF	BP	CC	MF	BP	CC	MF	BP	CC
DeepGOPLUS(improved)	0.590	0.604	0.579	0.244	0.231	0.163	1.961	2.422	1.918	0.113	0.075	0.075
DeepGOSE	0.587	0.606	0.578	0.240	0.234	0.178	1.959	2.406	1.915	0.111	0.083	0.081
**MAGIN-GO**	**0.653**	**0.728**	**0.615**	**0.324**	**0.301**	**0.203**	**2.078**	**1.922**	**1.896**	**0.215**	**0.114**	**0.172**
MEGA-GO	0.772	0.859	0.753	0.457	0.426	0.314	1.942	1.893	1.711	0.305	0.196	0.245
MMSNet	0.804	0.881	0.749	0.449	0.420	0.307	1.910	1.961	1.678	0.312	0.193	0.252

Table notes: Arrows (↑) indicate higher is better; (↓) indicate lower is better.

### Error analysis

This experiment systematically analyzes the errors of multiple functional prediction models (including MAGIN-GO, MMSNet [22], DeepGO-SE [15], etc.) based on the frequency of GO terms in the training set and their depth within the ontology structure. The experiment calculates the depth of each GO term using breadth-first search starting from three root nodes. Simultaneously, the frequency of each term is obtained by counting its occurrence in the training labels. During evaluation, the F-max is independently computed for each GO term in the test set to derive the optimal performance metric for each term. Terms were then categorized into three frequency groups: high-frequency (≥100 occurrences), medium-frequency (10–99 occurrences), and low-frequency (<10 occurrences). They were further grouped by depth into shallow-level (0–3), medium-level (4–6), and deep-level (7–20) categories. The average F-max score was calculated for terms within each group.

**Fig 4:**
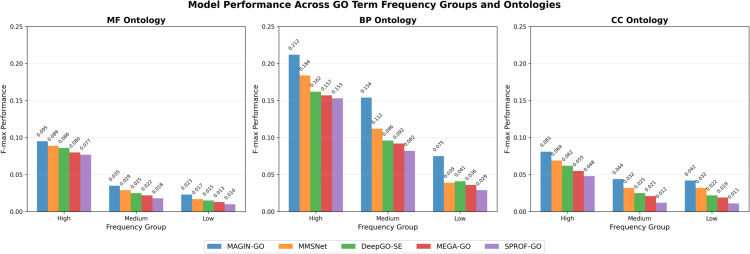
GO term frequency on three ontologies.

Results in Fig 4 demonstrate that MAGIN-GO achieved the highest performance across all categories, particularly attaining an F-max of 0.212 in the BP high-frequency group. This represents a nearly 40% improvement over other models like SPROF-GO [20], which achieved 0.153 in the same group. More critically, MAGIN-GO also demonstrated significant advantages in the more challenging low-frequency groups: for the BP ontology, its low-frequency F-max reached 0.075, while comparative methods like MEGA-GO [21] and SPROF-GO achieved only 0.048 and 0.051 respectively, representing a relative improvement exceeding 47%. Similar trends were observed in the low-frequency groups of MF and CC, with improvement rates generally ranging between 30%–50%. These results indicate that MAGIN-GO not only excels in predicting common functions but, more importantly, demonstrates stronger generalization capabilities for rare function prediction. This may stem from its deep integration of multi-source information (e.g., sequence, structure, interaction networks) and effective modeling of the GO ontology hierarchy.

This analysis highlights the limitations of current methods in predicting rare and highly specific functions, providing clear directions for future work, including incorporating hierarchical losses, graph neural network modeling, or data augmentation strategies tailored to long-tail problems.

### Explainability analysis

**Fig 5:**
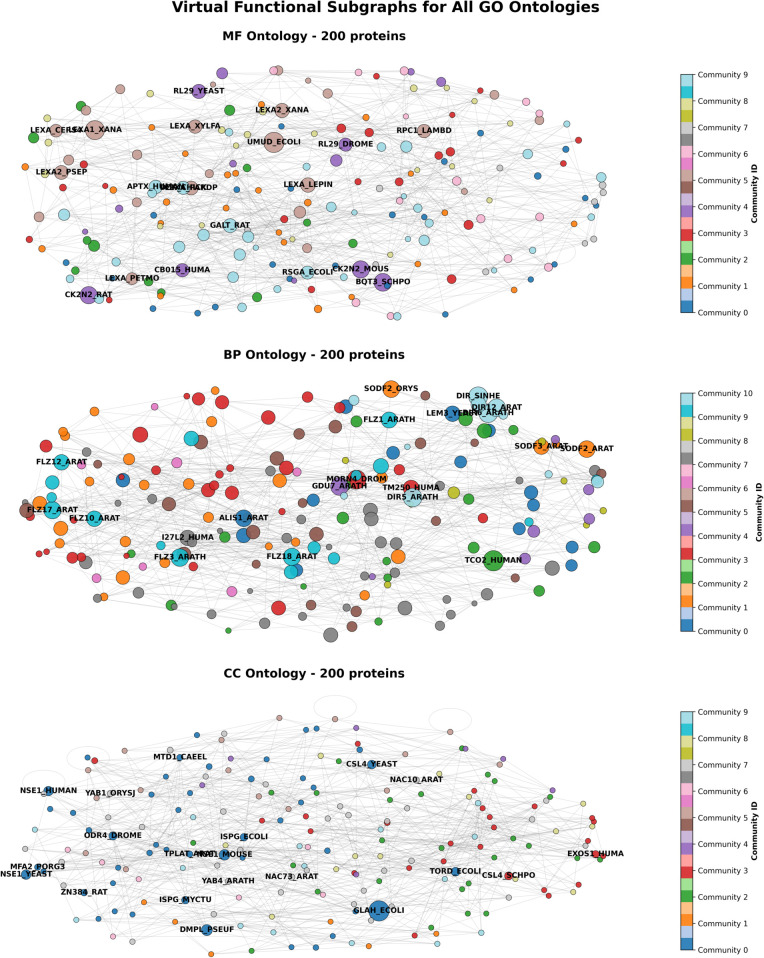
Virtual functional subgraph analysis on MF, BP, and CC.

We introduce the Virtual Functional Subgraph experiment to provide an intuitive interpretability analysis of MAGIN-GO’s prediction results. As shown in Fig 5, this visualization constructs an abstract network based on functional semantic similarity, revealing functional associations and modular structures among proteins across different GO domains. The three subgraphs correspond to the MF, BP, and CC domains, each containing 200 protein nodes from the test set. Node size reflects model importance scores, while color represents functional communities identified by the Louvain algorithm, clearly illustrating the organizational patterns of protein functions.

Through deep integration of multi-source information, MAGIN-GO enables the model to transcend traditional homology transfer or single-feature learning, establishing connections directly within the functional semantic space. The formation of communities in the figure embodies this mechanism: they are not based on physical interactions but on functional similarity reflected by ESM-2 sequence embeddings, clustered via the Louvain algorithm. Thus, each community represents a highly cohesive module within the functional semantic space, where proteins share similar biochemical activities, participate in analogous biological processes, or localize to comparable cellular components.

Specifically, within the MF subgraph, we observe multiple well-defined communities, such as those represented by purple (Community 4) and light blue (Community 9). Proteins within these communities, such as RL29_YEAST and LEXA_XANA, suggest potential membership in specific functional families (e.g., the LEXA transcription factor family). This demonstrates that the MAGIN-GO model successfully aggregates proteins with similar molecular functions, even across different species. This clustering based on functional semantics rather than species origin demonstrates the model’s robust generalization capability and validates the paper’s conclusion that “incorporating GO structural information effectively enhances MF prediction performance.” Within the BP subgraph, community structures become more complex and intertwined, exemplified by extensive cross-connections between the orange (Community 1) and red (Community 3) communities. This reflects the intrinsic nature of biological processes—a complex biological process often involves the coordinated action of multiple molecular functions and cellular components, with blurred functional boundaries and high dynamism. The model performs relatively weakly in this domain (Fmax=0.458), and the intricate community network structure in the graph precisely corroborates this: the lack of clear boundaries between functional modules increases prediction difficulty. Finally, within the CC subgraph, community segmentation appears relatively loose with dispersed node distribution. This aligns with CC ontology achieving the highest AUPR (0.754) and Fmax (0.752) in the paper, indicating that cellular component functions typically exhibit stronger spatial specificity. Their functional definitions are relatively well-defined and distinguishable, enabling the model to learn clearer classification boundaries and form structurally more independent communities.

### Case study

Finally, to further evaluate the predictive capabilities of our model, we employed MAGIN-GO for functional prediction of human and yeast proteins. It is important to emphasize that our research focuses not only on the model’s performance itself, but also on its practical application value in real biological scenarios.

We selected two proteins with well-studied functions: P63279 (UBC9_HUMAN), a human ubiquitin ligase involved in protein modification and cell cycle regulation; and P02381 (RMAR_YEAST), a yeast protein participating in nucleic acid metabolism. As shown in Table 10, MAGIN-GO successfully predicted all experimentally validated GO terms for these proteins, demonstrating high recall and precision in capturing known functional annotations.

**Table 10:** Examples of yeast and human protein function prediction.

Protein	Real Function	Predicted Function
P63279 (UBC9_HUMAN)	GO:0009058 GO:0016070	GO:0009058 GO:0016070
	GO:0006139	GO:0006139
	GO:1901576	GO:1901576
	GO:0008152	GO:0008152
	GO:0065007	GO:0065007
	GO:0010467	GO:0010467
	GO:0019538	GO:0019538
	-	GO:0006396
	-	GO:0006766
	-	GO:0044283
	-	GO:0006399
P02381 (RMAR_YEAST)	GO:0003824	GO:0003824
	GO:0043604	GO:0043604
	GO:0044267	GO:0044267
	GO:0008152	GO:0008152
	GO:0006807	GO:0006807
	GO:0009987	GO:0009987
	GO:0044238	GO:0044238
	-	GO:0019538
	-	GO:0010137
	-	GO:0008218
	-	GO:0097176
	-	GO:0035212

Notably, beyond fully reproducing existing ground truth labels, MAGIN-GO proposed novel GO annotations not present in the original benchmark datasets. For example, for UBC9_HUMAN, the model predicted GO:0006396 (RNA processing) and GO:0006399 (tRNA metabolism), which have reasonable biological relevance to its known post-translational modification and cellular homeostasis regulation roles. Similarly, for RMAR_YEAST, the model newly predicted terms such as GO:0010137 (pseudouridine synthesis) and GO:0097176 (rRNA methylation), suggesting the protein may participate in RNA modification pathways—a conjecture supported by recent research literature on ribosomal RNA processing.

## Conclusion

In this paper, we propose MAGIN-GO—an end-to-end deep learning framework for protein function prediction. The core contribution of this work lies in innovatively integrating three key information sources within a unified dual-graph neural network architecture: capturing the local topology of PPI networks via GIN, modeling global sequence and contextual dependencies through GMSA, and incorporating structured GO semantic information via Anc2Vec embeddings. Comprehensive evaluation on the UniProtKB/Swiss-Prot dataset demonstrates that MAGIN-GO outperforms current state-of-the-art methods, while ablation experiments validate the indispensability of each component. By effectively capturing and integrating multi-scale, multi-source biological information, MAGIN-GO provides a powerful and precise tool for protein function exploration.

MAGIN-GO has been released as open source on GitHub (https://github.com/sick-hasg/artical.git). Additionally, the dataset used in this project is available at (https://deepgo.cbrc.kaust.edu.sa/data/deepgo2/training-data.tar.gz).

In our future work, we will continue to investigate new methods to improve the prediction accuracy of MAGIN-GO. AlphaFold2 has made amazing achievements in protein 3D structure prediction. The distance maps generated from the tertiary structures predicted by Alphafold2 can capture the information of the alpha carbon atoms of the amino acids, and the distance maps can be used as an additional embedding in future research features to see if they can further improve the prediction accuracy. In addition, we plan to introduce more learnable features into the prediction methods in the future, such as a series of methods for multiple sequence alignment of target proteins to generate additional embedded features to see if they can further improve the prediction accuracy.

Another key direction is enhancing model robustness and extending its applicability to large-scale, unreviewed datasets such as UniProtKB/TrEMBL. We plan to explore computational methods for filling PPI networks to address proteins lacking manually reviewed interactions. Concurrently, we will develop a fallback mechanism enabling the model to maintain core functionality when PPI information is unavailable. These enhancements will significantly broaden our method’s applicability and practical value while effectively addressing current limitations in handling noisy, functionally uncharacterized protein sequences.
